# Ten Candidate Genes Were Identified to Be Associated with the Great Growth Differentiation in the Three-Way Cross Hybrid Abalone

**DOI:** 10.3390/ani15020211

**Published:** 2025-01-14

**Authors:** Qizhen Xiao, Shihai Gong, Zekun Huang, Wenzhu Peng, Zhaofang Han, Yang Gan, Yawei Shen, Weiwei You, Caihuan Ke, Xuan Luo

**Affiliations:** 1 Fujian Provincial Key Lab of Coastal Basin Environment, Fujian Polytechnic Normal University, Fuqing 350300, China; xiaoqizhen_fjsm@126.com; 2 State Key Laboratory of Mariculture Breeding, College of Ocean and Earth Sciences, Xiamen University, Xiamen 361102, China; shihaigong@stu.xmu.edu.cn (S.G.); zkhuang@stu.xmu.edu.cn (Z.H.); pengwz@stu.xmu.edu.cn (W.P.); zfhan@xmu.edu.cn (Z.H.); yanggan@stu.xmu.edu.cn (Y.G.); shenyawei216@stu.xmu.edu.cn (Y.S.); wwyou@xmu.edu.cn (W.Y.); chke@xmu.edu.cn (C.K.); 3 Abalone Research Center, Fujian Minruibao Marine Biotechnology Co., Ltd., Xiamen 361102, China

**Keywords:** three-way cross, abalone, GWAS, transcriptome, growth

## Abstract

Abalone is an economically important mollusk, whose slow growth has impeded the recovery of its wild populations and development of aquaculture. The three-way cross hybrid abalone ((*Haliotis discus hannai*♀ × *H. fulgens*♂)♀ × *H. gigantea*♂, DF × SS) demonstrated notable diversity in growth traits across the population with genetic differentiation, offering a model for exploring the molecular mechanisms of abalone growth. In this study, a total of 89 SNPs and 97 candidate genes were identified to be associated with growth-related traits of abalone using whole-genome resequencing and a genome-wide association study (GWAS) analysis. Then, ten overlap genes were found among these candidate genes by combining the results of GWAS and comparative transcriptomic analyses between the large individuals (L group) and small individuals (S group) of DF × SS. These overlap genes include up-regulated genes (*fabG*) and down-regulated genes (*HMCN1*, *TLR3*, *ITIH3*) between the L and the S groups, which are thought to function in growth in other organisms. The biological functions of these candidate genes in abalone still have to be confirmed, but they have improved our understanding of the molecular mechanisms behind abalone growth traits and provided molecular markers for abalone breeding programs.

## Simple Summary

As a key economic species, the breeding of new strains of abalone has been undertaken across various regions to address genetic resource degradation caused by the industry’s rapid expansion. Abalone grows slowly and must be cultured for several years to achieve marketable standards; thus, the slow growth rate is an important factor hindering the rapid expansion of the abalone aquaculture industry. The three-way cross hybrid abalone demonstrated notable diversity in growth traits across the population with genetic differentiation, offering a model for exploring the molecular mechanisms of abalone growth. In this study, we performed a genome-wide association study and transcriptome aimed at identifying growth-related traits in a three-way cross hybrid abalone. Ten candidate genes were identified to be associated with the great growth differentiation in the three-way cross hybrid abalone. The results of our findings will not only enhance our knowledge of the genetic mechanisms of growth traits in abalone but also supply useful single nucleotide polymorphisms for molecular marker-assisted selection and propagation of abalone.

## 1. Introduction

Growth traits are important for economically important aquatic species, as they directly affect production, and thus growth-related traits are the main foci of most selective breeding programs. Most of the growth-related traits known are quantitative traits and are influenced by several micro-efficient genes and various environmental factors [1]. Thus, the search for the main effector genes and their utilization in selection are the important basis for the improvement of growth traits. Traditional breeding methods aimed at increasing growth rates rely on phenotypic assessment of families or individuals, which is sluggish and inefficient [2]. High-throughput sequencing technology has shown strong advantages in the screening of growth-related candidate genes and assisting molecular breeding because it is considered to be more accurate, stable, and practical [3]. Using genome-wide association analysis and transcriptome analysis, researchers have identified a series of SNPs and candidate genes related to growth traits in catfish [4], rainbow trout [5], *Argopecten scallops* [6]. The successful screening and localization of these new molecular markers have enriched the knowledge of growth regulation and also laid an important foundation for the accurate localization of molecular markers in aquatic species and the further development of molecular-assisted breeding in the future.

Abalone, a group of widespread marine gastropods, is regarded as one of the world’s most important fishery products [7]. The decline in abalone fisheries productivity and the depletion of wild abalone resources have led to the global development of abalone aquaculture, particularly in China, where almost 90% of the world’s abalone aquaculture production occurs [8]. On the one hand, abalone aquaculture has been severely affected by disease outbreaks and high water temperatures, leading to high levels of mortality [9]. To solve these problems, extensive research on abalone crossbreeding has been carried out in China, and crossbreeding technology has served an important role in enhancing abalone production during the last two decades [10,11,12]. On the other hand, the sluggish growth rate of abalone is a major problem impeding the quick expansion of the abalone aquaculture industry, as abalone requires many years to reach commercial standards [13]. Therefore, research on the genetic mechanisms underlying abalone growth-related features is still lacking, even though understanding their molecular pathways is crucial for increasing abalone output.

Crossbreeding is an effective method of genetic improvement. According to the classification of crosses, hybrids include single-cross hybrids, three-way cross hybrids, and double-cross hybrids. Of these, the three-way cross hybrids are produced by a single-cross hybrid and a homozygous population. Compared to single-cross hybrids, such three-way cross hybrids have exhibited moderate levels of production potential but good adaptability to environmental variation due to their abundant genetic resources [14]. The application of three-way cross hybrids has been widely used in livestock and crop research [15,16,17,18], but has rarely been reported in aquatic animals. In abalone, the previous research has shown that the three-way crosses hybrids display heterosis for growth performance, heat tolerance, and hypoxia tolerance [19]. The progeny of the three-way cross abalone exhibited trait segregation on the mantle color, and GWAS analysis showed that a genomic region on chromosome 15 was associated with mantle color [20]. In addition, previous land-based culture experiments have shown that the three-way cross hybrid abalone ((*Haliotis discus hannai*♀ × *H. fulgens*♂)♀ × *H. gigantea*♂, DF × SS) displayed genetic differentiation in growth traits. Under the same rearing conditions, the proportion of large-sized individuals (24.81%) and small-sized individuals (34.58%) in the three-way cross hybrid abalone population was both relatively high at the adult stage. Specifically, the distinction between the largest and smallest individuals was 2.86 times in shell length and 24.64 times in body weight [19]. Therefore, in-depth studies on interspecies, the three-way cross hybrid abalone, which are characterized by growth differentiation differences, can provide new ideas and techniques for genetic breeding of shellfish.

In this study, we conducted a genome-wide association study (GWAS) analysis of eight growth-related traits (shell length, shell width, total weight, shell weight, foot muscle weight, the ratio of shell length and shell width, the ratio of foot muscle weight and wet weight, and the ratio of wet weight and shell length) in the three-way cross hybrid abalone to identify growth-associated SNPs and candidate genes. Then, comparative transcriptome analysis was conducted between large individuals (L group) and small individuals (S group) of DF × SS. Furthermore, candidate genes associated with growth in the GWAS analysis were compared with differentially expressed genes (DEGs) in the transcriptome analysis. Furthermore, candidate genes associated with growth in the GWAS analysis were compared with differentially expressed genes (DEGs) in the transcriptome analysis, and the overlap was identified as key genes. This study will provide molecular markers for breeding fast-growing abalone strains, and the results will be of great practical significance for the transformation and advancement of the abalone culture industry. Also, this study will contribute to our understanding of the molecular mechanisms of growth traits in abalone.

## 2. Materials and Methods

### 2.1. Material Construction

The three-way cross hybrid abalone, DF × SS ((*H. discus hannai*♀ × *H. fulgens*♂)♀ × *H. gigantea*♂), was established at the Fuda Abalone Farm (Jinjiang, China). After 14 months of culturing in the same environment, a total of 115 individuals of DF × SS were randomly collected for measuring eight growth-related traits, including five measurable traits (shell length, *SL*; shell width, *SW*; total weight, *TW*; shell weight, *TS*; and foot muscle weight, *TM*) and three calculable traits (the ratio of shell length and shell width, *LW*; the ratio of foot muscle weight and wet weight, *MR*; and the ratio of wet weight and shell length, *F*).

All traits data were checked for outliers and normality using the Shapiro–Wilk test implemented in SPSS v24.0, and traits that were not normally distributed were transformed by normal scores using Blom’s formula. Ratio variables were log-transformed to obtain a normality distribution. Pairwise Pearson correlation coefficients were calculated between growth-related traits using SPSS v24.0.

### 2.2. DNA Extraction and Sequencing

A total of 115 14-month-old individuals of three-way cross hybrid abalone with various growth traits were collected from Fuda Abalone Farm (Jinjiang, China). The foot muscle tissues were dissected and immediately frozen in liquid nitrogen for DNA extraction. Genomic DNA extraction from foot muscle samples using the DNeasy 96 Kit (Qiagen, Shanghai, China). The quality and quantity of DNA were evaluated using agarose gel electrophoresis and Nanodrop2000 (Thermo Scientific, Wilmington, DE, USA), respectively. Paired-end libraries with an insert size of 350 bp were constructed for the DNA samples and sequenced on the Illumina NovaSeq 6000 platform (Illumina, San Diego, CA, USA) using the 150 bp paired-end strategy.

### 2.3. SNP Discovery and Genotyping

The quality control of sequence reads for each sample was conducted using an in-house Perl script. The following reads were removed: reads aligned to the barcode adaptor, reads with ≥10% unidentified nucleotides (N), and low-quality reads (where >50% of the bases have a phred quality score ≤ 20). The clean reads were aligned to the *H. gigantea* genome (unpublished) by the BWA mem tool v0.7.11 with default parameters of the software [21]. A set of raw SNPs was generated by SAMtools software v1.17 [22] for variant detection and filtering of the generated BAM files. Further quality control of the raw SNPs was performed using Vcftools v0.1.16 [23] in the following steps: (1) Delete all non-biallelic SNPs; (2) The following SNPs and samples were excluded: average sequencing depth < 8 × (-minDP), SNPs with minor allele frequency (-maf) < 0.05, SNPs with a deviation from Hardy–Weinberg equilibrium < 0.001 (-hwe), and individuals with a missing data rate (-mind) > 0.2.

### 2.4. GWAS Analysis for Growth-Related Traits of Abalone

In order to reduce the false-positive rate, principal components analysis (PCA) based on SNPs was conducted by PLINK software v1.9 [24]. The GAPIT software v3.0 [25] was used for association analysis, where the *SL*, *SW*, *TW*, *TS*, *TM*, *LW*, and *F* traits were calculated using the FarmCPU method, and the *MR* trait was calculated using the Blink method. GWAS analysis was performed on SNP loci collected from 115 individuals. The threshold *p*-value for genome-wide significance was calculated based on the number of qualified markers applying the Bonferroni correction. When an SNP scored lower than the significance cutoff (1 × 10^−5^), the sequence of each marker was extracted and put as a query for BLASTN to find the *H. gigantea* genome. Manhattan plots were performed with the R CMplot package v4.5.1, and such putative SNPs were captured (*p* < 1 × 10^−5^) for subsequent bioinformatics analyses. In addition, based on the reference genome annotation information, the gene in which the significantly associated SNP was located or the closest gene upstream or downstream was used as a candidate gene associated with growth of abalone.

### 2.5. Transcriptomic Analysis

From the 115 GWAS-sequenced individuals, we selected two groups that showed extreme values in growth-related traits as transcriptome samples. Three large individuals (L group, the average shell length and total weight were 67.20 mm and 39.13 g, respectively) and three small individuals (S group, the average shell length and total weight were 27.77 mm and 2.68 g, respectively) from the three-way cross hybrid abalone were dissected, and eight growth-related traits were measured (Table S1). The foot muscle tissues were placed in liquid nitrogen immediately after collection and then transferred to a −80 °C freezer for storage. Total RNA was extracted by the Trizol method, and the purity and integrity of RNA were checked. Library preparation and sequencing were completed by Novogene (Beijing, China). RNA-seq libraries were constructed and sequenced on the HiSeq platform (Illumina, USA) to generate 150-nucleotide paired-end reads.

Raw reads were filtered using fastp v0.19.4 [26]. The clean reads were aligned to the *H. gigantea* genome using HISAT2 v2.2.1 [27], generating BAM files. The BAM files were sorted by using SAMtools v1.17 and then used to calculate abundances of annotated genes in the *H. gigantea* genome using StringTie v2.1.7 [28]. The gene read counts were extracted with the pre-pDE.py script in StringTie. The gene expression profiles were derived by the TMM normalization of the read counts with an rlog transformation using the R package DESeq2. The differentially expressed genes (DEGs) were identified using the criteria of a false discovery rate (*FDR*) < 0.05 and |log2FoldChange| > 1. R package pheatmap [29] was used to show the expression levels of these genes. The Gene Ontology (GO) and Kyoto Encyclopedia of Genes and Genomes (KEGG) pathway enrichment analyses of genes were performed using the R package clusterProfiler [30] with a modified Fisher’s exact test based on *p* < 0.05 and *FDR* cutoff < 0.05.

## 3. Results

### 3.1. Descriptive Statistics for Growth-Related Traits

**Figure 1:**
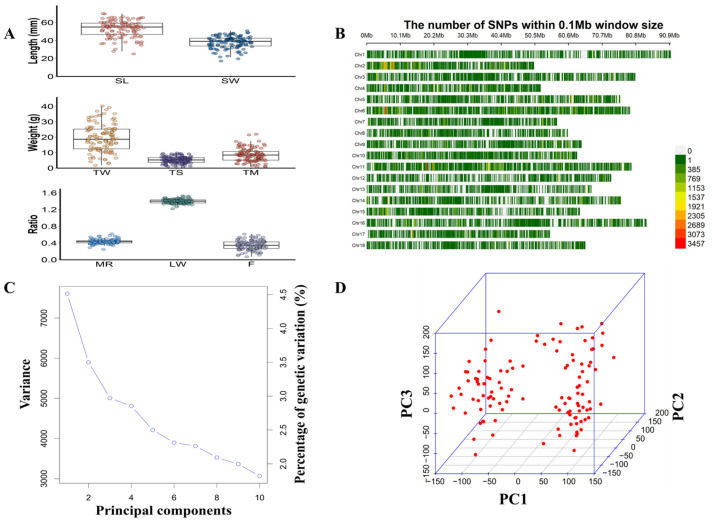
Growth-related traits and genetic variants distribution of individuals used for GWAS. (**A**) Box plots of eight growth-related traits of 115 individuals of three-way cross hybrid abalone (DF × SS). (**B**) Distribution of SNPs on each chromosome (the number of SNPs within a 0.1 Mb window size). (**C**) The gravel plot in principal component analysis (PCA). (**D**) 3D PCA plot.

Summary statistics for all growth-related traits are listed in Figure 1A. The mean values of *SL*, *SW*, *TW*, *TS*, *TM*, *MR*, *LW*, and *F* were 52.88 ± 9.10 mm, 37.86 ± 6.79 mm, 19.47 ± 8.77 g, 5.30 ± 2.11 g, 8.57 ± 4.22 g, 0.43 ± 0.06, 1.40 ± 0.05, and 0.35 ± 0.11, respectively. Shapiro–Wilk tests indicated that four growth-related traits (*TW*, *TS*, *LW*, and *F*) were normally distributed (*p* > 0.05). However, four other growth-related traits (*SL*, *SW*, *TM*, and *MR*) were non-normally distributed (*p* < 0.05), and thus these traits were transformed to normal values (Figure S1). Pearson correlation coefficients between the eight growth-related traits ranged from −0.33 to 0.99 (Table S2). Pairwise positive correlation coefficients (0.91–0.99) between the six measurable traits (*SL*, *SW*, *TW*, *TS*, *TM*, and *F*) were high. Pairwise correlation coefficients (−0.33 to 0.42) between the three calculable traits (*LW*, *MR*, and *F*) were low. The high correlation between these traits suggests that the same genes, loci, or genomic regions may exist to control the growth traits of the studied three-way cross hybrid abalone.

### 3.2. Quality Control of Sequencing Data

Whole-genome resequencing was performed on 115 individuals, producing a total of 1545.16 Gb of raw data. After deleting the low-quality reads, 1502.18 Gb of clean data were available. The average sequencing depth per sample was 9.28× since the total length of the assembled *H. gigantea* reference genome was approximately 1.4 Gb. After controlling for quality, there were 746,308 SNPs and 115 samples that were available for further analysis. The average marker density across the genome was approximately 1.69 kb/SNP (Figure 1B).

### 3.3. GWAS of Growth Traits

The gravel plot shows that the proportion of genetic variation explained with the first four PCs (13.84%) was significantly higher than those with other PCs (12.99%) (Figure 1C). The 3D PCA plot also shows that PC1 and PC2 could stratify the samples (Figure 1D), and thus in the subsequent GWAS analysis, PC1 and PC2 were added as covariates to the model to reduce false positives and correct for the effect of sample stratification on the association analysis.

**Figure 2:**
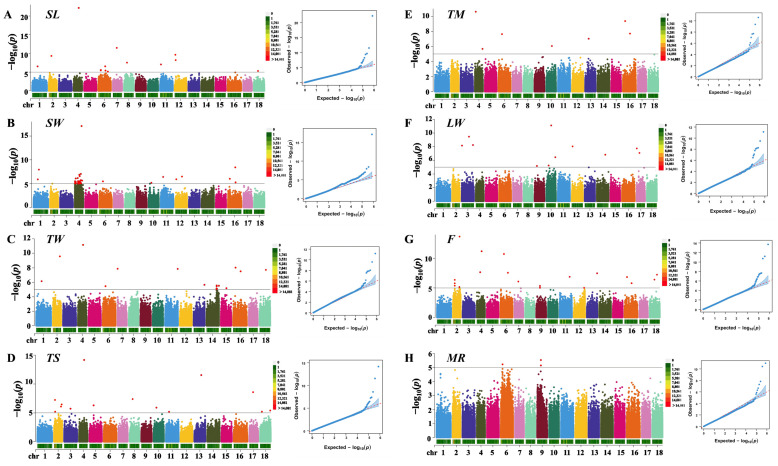
Manhattan plots and QQ plots of (**A**) *SL*, (**B**) *SW*, (**C**) *TW*, (**D**) *TS*, (**E**) *TM*, (**F**) *LW*, (**G**) *F*, and (**H**) *MR*. The black line represents the genome-wide significance threshold (−log10*P* = 5). The horizontal bars represent marker density on each chromosome. *SL:* shell length; *SW:* shell width; *TW:* total weight; *TS*: shell weight; *TM*: foot muscle weight; *LW*: the ratio of shell length and shell width; *MR*: the ratio of foot muscle weight and wet weight; *F*: the ratio of wet weight and shell length.

The GWAS was performed by utilizing 746,308 SNPs and 115 samples from three-way cross hybrid abalone. After carrying out Bonferroni correction and applying the cutoff (*p* < 1 × 10^−5^), a total of 89 genome-wide SNPs significantly associated with growth-related traits were detected (Figure 2 and Table S3). In detail, four SNP loci showed significant effects on several traits: chr4_31036063 was significant for *TM*, *TW*, *TS*, and *SL* traits; locus chr16_45749077 was significant for the *F*, *SW*, and *TW* traits; locus chr16_47618889 was significant for the *SL* and *SW* traits, and locus chr13_42736357 was significant for the *TM* and *TS* traits. A total of 97 candidate genes were located and annotated according to 89 significant SNPs (Table S4), including calponin-2 (*Cnn2*), obscurin (*Obscn*), Wiskott–Aldrich syndrome protein family member 3 (*WASF3*), hemicentin-1 (*HMCN1*), perlucin, and kyphoscoliosis peptidase (*Ky*).

### 3.4. The Result of Transcriptomic Analysis

**Figure 3:**
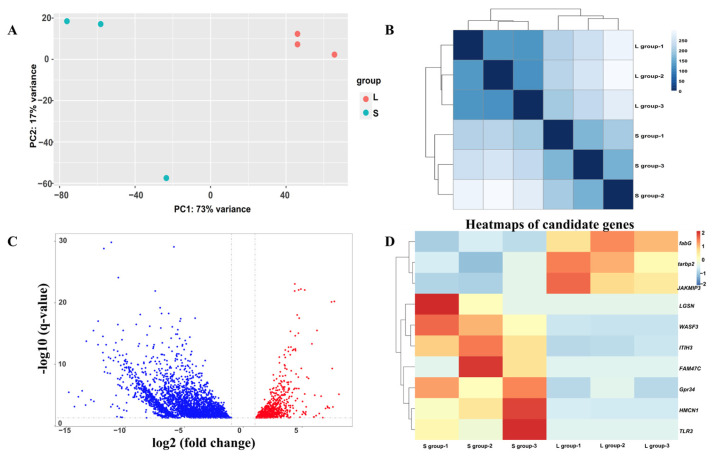
Comparative transcriptomic analysis of L group and S group in DF × SS. (**A**) PCA plot of transcriptome of abalone muscle samples. (**B**) Genome-wide clustering of foot muscle samples. (**C**) Volcano plot of gene expression in the muscle of the L group and the S group in DF × SS. The up-regulated and down-regulated differentially expressed genes (DEGs) are shown in red and blue dots, respectively. (**D**) Heatmap of overlap genes between GWAS candidate genes and differentially expressed genes in the transcriptome analyses.

The results of the transcriptome showed that an average of 22.28 million raw reads and 20.21 million pure reads were obtained from each sample by RNA-seq (Table S5). The PCA based on genome-wide gene expression profiling suggested that the two groups were notably separated in the PC1/PC2 score plot (Figure 3A), where PC1 explained 73% and PC2 explained 17% of the total variance. Sample clustering based on genome-wide expression analysis also clearly differentiated the samples, with three samples clustered into one class in the L group and three samples in the S group clustered into one class (Figure 3B).

**Figure 4:**
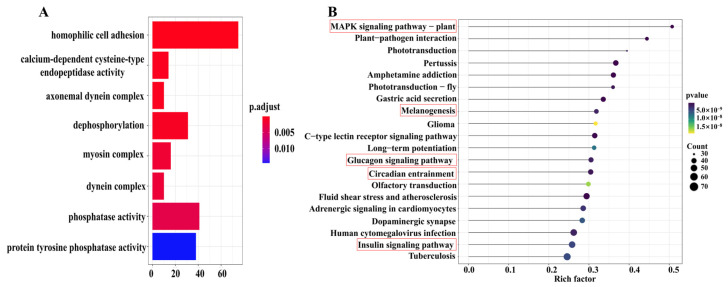
(**A**) GO and (**B**) KEGG pathway enrichment analysis for all DEGs.

A total of 3665 differentially expressed genes were identified between the L group and S groups. Of these, 705 genes were up-regulated and 2960 genes were down-regulated in the L group relative to the S group (Figure 3C). These DEGs were enriched in eight GO terms, including homophilic cell adhesion, axonemal dynein complex, calcium-dependent cysteine-type endopeptidase activity, dephosphorylation, myosin complex, dynein complex, phosphatase activity, and protein tyrosine phosphatase activity (Figure 4A). The KEGG pathway enrichment analysis showed that 3665 DEGs in the two groups were enriched in the MAPK signaling pathway (35 genes), circadian entrainment (49 genes), glucagon signaling pathway (47 genes), melanogenesis (43 genes), and the insulin signaling pathway (64 genes) (Figure 4B).

### 3.5. Integrated GWAS and Transcriptome Analysis

**Figure 5:**
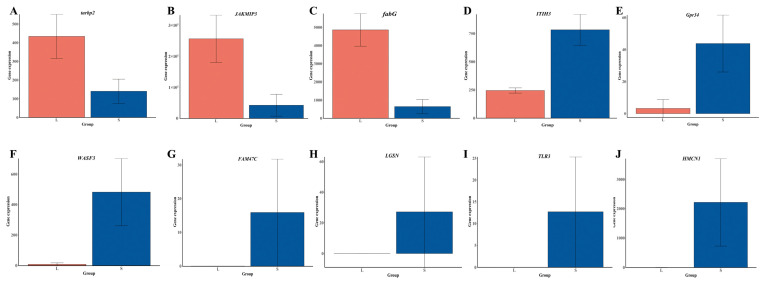
Expression of ten overlap candidate genes by RNA-seq in the L and S group. (**A**): *tarbp*; (**B**): *JAKMIP3*; (**C**): *fabG*; (**D**): *ITIH3*; (**E**): *Gpr34*; (**F**): *WASF3*; (**G**): *FAM47C*; (**H**): *LGSN*; (**I**): *TLR3*; (**J**): *HMCN1*.

A total of ten overlap genes were found between GWAS results and DEGs in transcriptome analysis (Table 1 and Figure 3D). Among them, three genes were up-regulated in large individuals of DF × SS, including 3-oxoacyl-[acyl-carrier-protein] reductase FabG (*fabG*), RISC-loading complex subunit tarbp2 (*tarbp2*), and janus kinase and microtubule-interacting protein 3 (*JAKMIP3*). However, seven genes were down-regulated in large individuals of DF × SS, including hemicentin-1 (*HMCN1*), lengsin (*LGSN*), Wiskott–Aldrich syndrome protein family member 3 (*WASF3*), putative protein FAM47C (*FAM47C*), toll-like receptor 3 (*TLR3*), probable G-protein coupled receptor 34 (*Gpr34*), and inter-alpha-trypsin inhibitor heavy chain H3 (*ITIH3*). The sequences of the ten overlap genes are shown in Table S6. Expression of ten overlap candidate genes by RNA-seq in the L and S groups is shown in Figure 5. There was a significant difference in the expression of these candidate genes in the L and S groups. This suggests that these candidate genes may play an important role in the growth traits of the three-way cross hybrid abalone.

## 4. Discussion

### 4.1. Genetic Differentiation Is the Most Important Feature of the Three-Way Cross Hybrid Abalone

Distant hybridization can rapidly combine the genetic material of the parents, resulting in significant changes in the appearance and molecular genetics of the hybrid progeny. In the formation of hybrids, genomes of different origins are recombined and exchanged, which facilitates the transmission of genetic variation to the offspring [31]. Genotypic changes often lead to phenotypic changes, making hybrid offspring show hybrid dominance in appearance, body size, growth rate, and disease and stress resistance [32]. Therefore, analyzing the genetic characteristics of hybrids is of great theoretical significance for crossbreeding. The interspecies three-way cross hybrid abalone combine the genomes of three abalone species (*H. fulgens*, *H. discus hannai,* and *H. gigantea*) and therefore show different degrees of genetic differentiation in various traits. For example, the offspring of the three-way cross abalone showed segregation of the mantle color trait, with some of the offspring having black markings on the mantle and some of the offspring lacking mantle markings [20]. And previous studies have found that the three-way cross abalone also show genetic differentiation in heat tolerance, with individuals with mantle markings being more heat-tolerant than those without mantle markings [20]. Under the same breeding environment, the difference between the largest and smallest individuals in the shell length of three-way cross hybrid abalone was 2.86 times. Also, in body weight, the difference between the largest and smallest values reached 24.64 times [19]. In this study, the coefficients of variation for growth-related traits were large in the three-way cross hybrid abalone, especially for *TM*, *TW,* and *TS*, which reached 0.49, 0.45, and 0.40, respectively (Figure 1A). This also proves that there is indeed a great genetic differentiation in the three-way cross hybrid abalone. Genetic differentiation is the most important feature of the three-way cross hybrid abalone, and analyzing the genetic characteristics of the three-way cross hybrid abalone has important theoretical significance and application value. However, due to the low fertilization rate of the three-way cross hybrid abalones [19], there are still some difficulties in the construction of experimental materials. An in-depth study of growth traits in reciprocal crosses (SS × DF and DF × SS) could better explain the reasons for genetic differentiation, for example, whether there is any influence of parental effects. In our future research, we will advance the seed production of three-way cross hybrid abalones to optimize the fertilization conditions and obtain sufficient experimental materials to further validate and consolidate our findings. Overall, the interspecies three-way cross hybrid can be used as the core stock for selection and breeding, making full use of the genetic differentiation and the characteristics of gonadal fertility to carry out selection and breeding of abalone strains with different traits as the parent abalone.

### 4.2. GWAS Is an Effective Technique to Elucidate the Genetic Variation Associated with Complex Growth-Related Traits in the Three-Way Cross Hybrid Abalone

Growth-related traits are important for economically important aquatic species, as they directly affect production, and thus growth-related traits are the main foci of most selective breeding programs. Most of the growth-related traits known are quantitative traits and are controlled by several micro-efficient genes in the genome. GWAS is an effective technique to elucidate the genetic variation associated with complex quantitative traits and generate markers and candidate genes for breed programs [33]. The genetic basis of growth traits in aquatic species has been extensively researched, and many key functional genes have been identified. For example, a genome-wide association analysis of growth-related traits in juvenile farmed Atlantic salmon revealed that growth traits were controlled by multiple micro-effective genes [34]. Jin et al. [35] applied GWAS to catfish traits and found that a locus in cluster 7 was strongly associated with head length, while the head width trait was located in cluster 9. Yu et al. [36] performed a GWAS study of growth-related traits in more than two hundred wild groupers, identifying body length-related SNPs and annotating 34 genes. Li et al. [4] investigated catfish body traits based on GWAS and finally located a genomic region of about 1 Mb on LG5, as well as a correlation between this genomic region and skeletal development. Zhou et al. [37] performed double digest restriction-site-associated sequencing on 220 large yellow croakers and finally identified 13 growth trait-associated SNPs distributed on eight chromosomes based on a GWAS analysis. This provided reliable genetic markers for understanding the genetic basis of growth and body size in the large yellow croaker population. Slow growth rate is an important factor hindering the rapid expansion of the abalone aquaculture industry, and thus increasing the growth rate is an important goal for the genetic improvement of abalone. In order to understand the genetic regulation mechanisms of growth traits in blotched snakehead (*Channa maculata*), Liu et al. [38] conducted a genome-wide association study on eight growth traits in 500 snakeheads, and the results showed that a total of 51 significant SNPs and 112 genes were identified. A previous GWAS of growth-related traits in the Pacific abalone identified several markers, providing a basis for exploring the regulatory mechanisms of abalone growth traits and enhancing the growth rate of abalone through gene editing and other methods [39]. Hybrid abalone have significant advantages in growth, but no GWAS analysis of growth-related traits has been reported. Previous experiments showed that three-way cross hybrid abalone are genetically differentiated for growth traits and therefore have the potential for further selection and breeding [19]. Thus, the three-way cross hybrids are excellent materials for studying the regulatory mechanisms of growth traits in abalone. In this study, a total of 89 SNPs were identified that were significantly associated with growth-related traits. A few of these SNPs were associated with multiple traits, suggesting the presence of coordinated regulation mechanisms. This result was consistent with the phenotypic correlation analysis of the eight traits (Table S2). All chromosomes in this study contained SNPs that were significantly associated with growth traits, and there were no significant regions of chromosomal clustering, suggesting that growth traits in ternary hybrids are controlled by multiple genes, similar to the findings of previous studies. For example, Peng et al. [39] conducted a GWAS analysis of 10 growth traits in the Pacific abalone and obtained 116 significant loci scattered across 18 chromosomes. Liu et al. [40] used RAPD, AFLP, and SSR markers to assess the location and impact of nine quantitative trait loci (QTL) for growth-related traits in Pacific abalone, finally detecting 28 significant QTLs (LOD > 2.4). However, all of the above studies were limited to the nuclear genome. The composition of the genetic structure of hybrids is complex, and therefore the trait characteristics of hybrids are also the result of the interaction of multiple factors. It has been shown that the formation of heterosis is related not only to the nuclear genome but also to the mitochondrial genome [41]. Hybrids usually have a more favorable genetic composition, the most efficient way of interaction of the nuclear genome and mitochondrial genome. Therefore, during the formation of hybrids, the nuclear and mitochondrial genomes are interacting with each other, giving the hybrids an advantage in gene expression and regulation during growth and development [42]. In this study, we investigated the growth-related traits of the three-way cross hybrid abalone based on the nuclear genome but failed to incorporate the mitochondrial genome for further analysis. In future studies, we will also take the mitochondrial genomes of the three-way cross hybrid abalones and their parents as one of the focuses of our research, such as their genetic structure and the correlation of hybrid advantage, in order to obtain more valuable research results.

### 4.3. Ten Candidate Genes Were Identified to Be Associated with the Great Growth Differences in the Three-Way Cross Hybrid Abalone

Functional gene discovery using high-throughput sequencing is a very effective tool in shellfish research. In the clam *Meretrix meretrix*, transcriptomic studies were performed on four different larval stages, resulting in the screening of differentially expressed genes related to development, growth, and shell formation, among others [43]. Qin et al. [44] conducted transcriptomic studies on *Crassostrea angulata* at eight different stages of growth and development and finally screened six functional genes related to growth and development, including epidermal growth factor receptor, adrenergic receptor, insulin-like growth factor 1 receptor, and follicle depressor precursor. Song et al. [45] analyzed the transcriptome of *Rapana venosa* at six different developmental periods and identified six genes related to neurosecretory function. Jiang [46] analyzed the transcriptomes of the top-selected, control, and bottom-selected groups of the Chinese pecten scallop “Nan’ao golden scallop” and screened six up-regulated genes associated with growth and development, of which three were associated with the regulation of *actinoglobulin* composition; one was involved in insulin production, one was involved in protein hydrolysis, and one was related to shell calcification. In order to investigate the molecular mechanisms of individual growth differences, large (L group) and small (S group) individuals in the DF × SS were analyzed in this study. The differences in growth between the two groups of individuals were evident under the same breeding environment (Table S1), and the two groups were also clearly distinguished by a genome-wide principal component analysis (Figure 3A) and sample clustering (Figure 3B). The intersection of the genes localized by GWAS and DEGs obtained from the transcriptome resulted in a final screening of 10 genes distributed across eight chromosomes (Table 1).

Among these candidate genes, *HMCN* is a highly conserved extracellular matrix protein that plays an essential function in cell growth, differentiation, migration, and stable cell–basement membrane contact [47]. Studies in *Caenorhabditis elegans* have found that the synthetic secretion of *HMCN* is concentrated in the muscle tissue of the body wall and the gonads [48]. Related studies in mice also found that *HMCN-1* is present in the extracellular matrix of the heart and plays a non-negligible role in the remodeling of the myocardium [49]. Peng et al. [39] identified a homologous gene, *HMCN2*, in the results of the GWAS analysis of 10 growth traits in Pacific abalone, and thus *HMCN1* may be instrumental in the growth and development of abalone. Yu et al. [50] identified gene *HMCN1* in the results of the GWAS analysis of feed efficiency in Pacific abalone, and *HMCN1* may affect feed efficiency ratio or residual feed intake traits through biological processes such as cell proliferation, nutrition metabolism, and the immune system. Thus, *HMCN1* may be associated with cell growth, differentiation, migration, and feed efficiency ratio in abalone.

*TLR*s are transmembrane proteins that are widely distributed in immune organs and tissues of the heart, brain, lungs, liver, and kidneys. The proteins play key roles in inflammation, immune cell regulation, and cell survival and proliferation. Song et al. [45] showed that there was a significant elevation of gene expression in the *TLR* signaling pathway in *Rapana venosa* after larval metamorphosis. Some studies have also shown that *TLR*s are known to take an active role in the early development of fish embryos and larvae. For example, the expression of *TLR3* and *TLR5* was elevated during larval hatching in a study of channel catfish and hybrid catfish [51]. Samanta et al. [52] studied the growth and development of Indian carp, rohu (*Labeo rohita*), and found that the expression of *TLR3* tended to increase after larval emergence from the membrane. Li et al. [53] found that the expression of *Mytilus coruscus* mussel *TLR* varied among developmental stages, and the highest expression of *TLR* genes was found in the juvenile stage of mussels after attachment metamorphosis, indicating that *TLR* may be involved in larval development.

*ITIH* is a glycoprotein with high inhibitory activity against trypsin that functions to stabilize the extracellular matrix [54]. The *WASP* family of proteins is instrumental in actin assembly in various cellular processes, including adhesion, vesicle transport, and migration [55]. The gene *fabG* is involved in the synthesis of fatty acids and has been linked to environmental tolerance, a factor that is essential for the survival of bacteria [56]. For example, *fabG* is an essential gene for the growth of *Sinorhizobium meliloti* [57]. The function of *Tarbp2* is mainly associated with immunity. In mouse studies, knockdown of *Tarbp2* resulted in growth defects and male sterility, suggesting an important role in development [58]. In grass carp (*Ctenopharyngodon idella*), *Tarbp2* can inhibit apoptosis by regulating protein kinase R phosphorylation through protein interactions, thereby inhibiting the activity of grass carp [59]. In this study, the expression of *Tarbp2* in the L group was significantly higher than that in the S group (Figure 3D), suggesting that *Tarbp2* may be involved in the growth of abalone and improve the immune ability. G protein-coupled receptors (*Gpcrs*) are a large group of membrane protein receptors and are the most abundant cell surface receptors. *Gpcrs* are responsible for the regulation of the immune system, the control of digestive functions, and the regulation of water homeostasis in the body [60]. For example, *Gpr54* plays an important role in the endocrine regulation of reproduction in fish, including feedback regulation of hormones and energy balance [61]. *Gpr34* is an integral membrane protein containing seven putative transmembrane structural domains. These proteins transmit signals into the cell interior by activating heterotrimeric G proteins, which in turn activate various effector proteins that ultimately lead to physiological responses [62]. *Gpr34* is involved in cell motility, differentiation, and mitosis. In this study, the expression of *Gpr34* in the S group was significantly higher than that in the L group (Figure 3D), suggesting that *Gpr34* may play an important role in the functional control of the digestive system and the regulation of water balance in abalone. *JAKMIP* (Jak and microtubule interacting protein) was identified for its ability to bind to the FERM (band 4.1 ezrin/radixin/moesin) homology domain of *Tyk2*, a member of the Janus kinase (Jak) family of non-receptor tyrosine kinases that are central elements of cytokine signaling cascades. *JAKMIP* 3 belongs to a family of three genes conserved in vertebrates and is predominantly expressed in neural tissues and lymphoid organs [63]. In terms of cellular metabolism, *JAKMIP3* may affect cellular metabolism through molecular interactions, or affect the transport and localization of key molecules in metabolic pathways. These effects may indirectly influence cellular energy metabolism, material synthesis and catabolism, and other processes. *LGSN* is a major protein of the vertebrate eye lens. It belongs to the hitherto purely prokaryotic glutamine synthetase I branch of the glutamine synthetase superfamily [64]. In zebrafish studies, *LGSN* specifically marks terminally differentiating lens fiber cells, and they suggest *LGSN* plays a role in fiber elongation or the establishment of fiber cell interactions [65]. The *FAM47C* protein structure has an amino acid length of 1035 residues and a mass of 115.3 kDa and is a member of the *FAM47* protein family. Studies have shown that *FAM47C* is associated with male fertility [66]. The detection of each of these candidate genes provides additional evidence and guidance for the study of the genetic mechanisms of growth traits in three-way cross hybrid abalone, but the details concerning functions and regulatory mechanisms require more in-depth experimental investigation.

## 5. Conclusions

In summary, we performed GWAS analyses for eight growth-related traits in three-way cross hybrid abalone, resulting in the identification of 89 significant SNPs associated with growth traits and the loci of 97 candidate genes. These genes included *Cnn2*, *Obscn*, *WASF3*, *HMCN1*, *Perlucin*, and *Ky*. Transcriptome analyses of two groups of individuals with significant growth differences identified 3665 differentially expressed genes. Finally, there were 10 genes distributed on eight chromosomes that overlapped between those identified by GWAS and those from the transcriptome analysis, including *HMCN1*, *TLR3*, *ITIH3*, and *fabG*. This study not only increases the understanding of the genetic mechanism of growth traits in abalone but also provides useful single nucleotide polymorphisms for molecular marker-assisted breeding of abalone. In addition, the data from our study can further enrich the basic biological theory of shellfish hybrids and provide important references for interspecific three-way cross hybrids, abalone breeding practices, and production application.

## Figures and Tables

**Table 1:** Functional annotation of candidate genes in the intersection of GWAS and transcriptome analyses.

Gene ID	SNP	Chromosome	Region	Start	End	Allele	Gene Name	Gene Annotation
HGI_T14376	Chr9_59504103	Chr9	intergenic	58006499	58126797	C/A	*HMCN1*	Hemicentin-1
HGI_T06123	Chr4_12106042	Chr4	intergenic	12090922	12092578	C/T	*LGSN*	Lengsin
HGI_T05584	Chr3_74188029	Chr3	intergenic	74333450	74399312	G/A	*WASF3*	Wiskott–Aldrich syndrome protein family member 3
HGI_T23406	Chr14_73608989	Chr14	intergenic	73669864	73675427	A/C	*FAM47C*	Putative protein FAM47C
HGI_T08489	Chr5_52996877	Chr5	intergenic	53019621	53021774	A/T	*TLR3*	Toll-like receptor 3
HGI_T16503	Chr11_18317868	Chr11	intergenic	18566930	18567951	C/T	*Gpr34*	Probable G-protein coupled receptor 34
HGI_T08344	Chr5_46100005	Chr5	intergenic	46079083	46097309	T/G	*ITIH3*	Inter-alpha-trypsin inhibitor heavy chain H3
HGI_T11050	Chr7_31718396	Chr7	intergenic	31668238	31677240	G/T	*tarbp2*	RISC-loading complex subunit tarbp2
HGI_T11923	Chr8_12170166	Chr8	mRNA	12062126	12172988	C/T	*JAKMIP3*	Janus kinase and microtubule-interacting protein 3
HGI_T06291	Chr4_17721291	Chr4	mRNA	17675906	17740990	C/T	*fabG*	3-oxoacyl-[acyl-carrier-protein] reductase FabG

## Data Availability

The sequencing reads for both whole genome-sequencing are archived in the China National GeneBank DataBase (CNGB) with BioProject accession CNP0003526, and the transcriptome sequencing are archived in CNGB with BioProject accession CNP0006550.

## Supplementary Materials

The following supporting information can be downloaded at: https://www.mdpi.com/article/10.3390/ani15020211/s1, Figure S1: Histogram distribution of eight growth traits in three-way cross abalone. Table S1: Phenotypic statistics of individuals sampled from the DF × SS for transcriptome; Table S2: Phenotypic correlation statistics for DF × SS; Table S3: Growth trait-related significant SNP loci; Table S4: The information of the candidate genes identified in the GWAS analysis; Table S5: Quality summary of original sequencing data. Table S6: The sequences of the ten overlap genes in three-way cross hybrid abalone.
