# Supplementary figures and images for: Ten Candidate Genes Were Identified to Be Associated with the Great Growth Differentiation in the Three-Way Cross Hybrid Abalone
Source: Animals (Basel). 2025 Jan 14;15(2):211. doi: 10.3390/ani15020211 (PMC11758661; doi:10.3390/ani15020211)

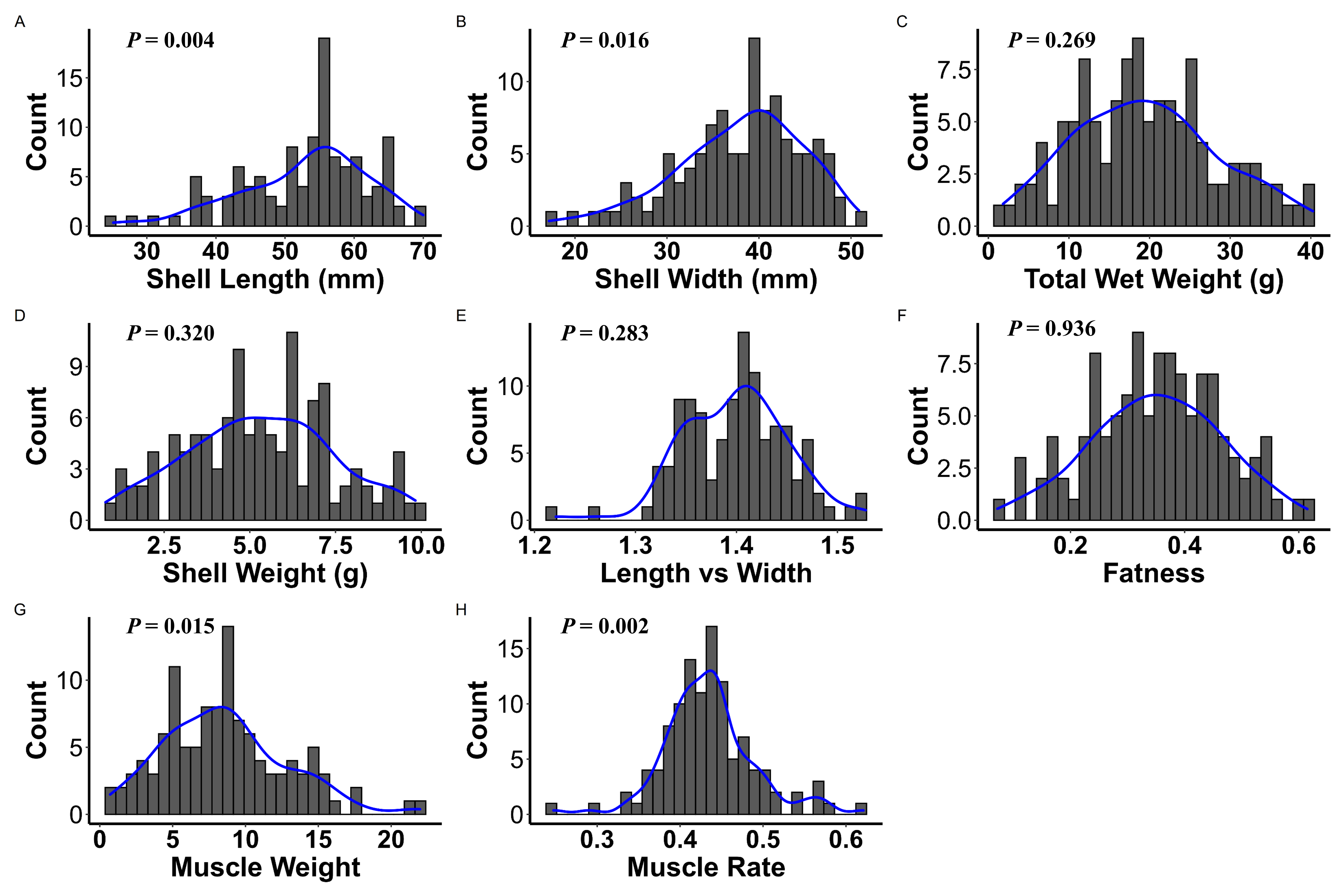

Supplement: Supplementary file 1 [file animals-15-00211-s001.zip › Figure S1.tif]
